# Human reliability assessment of intelligent coal mine hoist system based on Bayesian network

**DOI:** 10.1038/s41598-022-26493-4

**Published:** 2022-12-19

**Authors:** Linhui Sun, Liao Wang, Chang Su, Fangming Cheng, Xinping Wang, Yuanrui Jia, Ziming Zhang

**Affiliations:** 1 School of Management, Xi’an University of Science and Technology, Xi’an, 710054 China; 2 School of Safety Science and Engineering, Xi’an University of Science and Technology, Xi’an, 710054 China

**Keywords:** Health occupations, Risk factors, Energy science and technology, Engineering

## Abstract

The human reliability of intelligent coal mine hoist operation system is affected by many factors, in order to reduce the occurrence of human error in the hoist system and improve the reliability of the system. The characteristics of phased-mission task operation of hoists is combined, the phase dependence of human cognitive errors is considered and, a new human reliability evaluation method is proposed with the help of Bayesian network (BN) model in this paper. Firstly, the phase dependence of human cognitive errors was analyzed based on the cognitive behavior model. Then the human error analysis in the hoist system was carried out, and several main performance shaping factors are selected. Secondly, BN was used to build the human reliability model of the hoist system at each stage. Finally, it is found that the phase dependence of cognitive errors has a negative impact on the human reliability of the hoist system through the case analysis. At the same time, several main performance shaping factors (PSFs)were quantitatively analyzed by using the reverse reasoning ability of BN, which proves the effectiveness of the proposed method, and provides a scientific and reasonable theoretical basis for the development of effective human error prevention measures for the operation of intelligent coal mine hoists.

## Introduction

Coal mines are a safety accident prone industry, With the development of smart coal mines, the working conditions, automation of equipment and the production environment have been greatly improved^1,2^. The number of fatal coal mine accidents and the death rate of one million tons of coal decreased from 5670 and 5.28 in 2001 to 228 and 0.059 in 2020, respectively^3^. Even if the adoption of digital technologies has lessened the frequency of mishaps in industrial systems, accidents brought on by human mistake are still occurring at a high rate^4^. This is true since incorporating new technology into the growth of intelligence also creates a number of issues^5^. First, the volume of information in digital systems has substantially expanded, making it more challenging to find the needed data. Second, unlike conventional coal mines, intelligent coal mine systems have both primary and secondary jobs. The number of secondary activities is significantly higher than the number of primary tasks, and secondary tasks add to the operator's workload. The development of intelligent coal mines raises the uncertainty of human relative to conventional coal mines. The probability of Human error probability (HEP) is also increased by complicated procedures^6^. The coal mine hoist, as the monitoring and operation center of the production material and operator transportation system of the coal mine enterprise, is an important channel to link up and down the coal mine^7^. Coal mine hoist as a coal mine enterprise production materials, operators transport system monitoring and operation center, is an important link between the coal mine shaft underground, the safe operation of the hoist is an important link to ensure the safe production of coal mines. During the continuous development of intelligent coal mines, the reliability of the equipment is continuously improved, and it is more necessary to focus on human reliability analysis and assessment, so as to improve the overall reliability of the system. Human reliability assessment is of great significance to improve the safety and efficiency of intelligent coal mine hoist systems as well as to prevent human error accidents^8^.

Increasingly more operators are transitioning from manual operators to machine system supervisors in the context of intelligence and digitalization^9^. A large number of cognitive activities are involved during multiple phase operation tasks in intelligent coal mine hoist systems. The occurrence of cognitive mistakes is not only related to the cognitive activity associated with the present phase, but it is also influenced by the results of the cognitive activities associated with the previous phase, it is crucial to keep in mind when undertaking human reliability analysis. For instance, if the hoist operator does not observe the instrument readings, he/she may make an error. Hoist operators usually experience several phases while performing their tasks, and the human reliability of phased-mission systems (PMSs)needs to be analyzed, and the interdependence between phases, which is natural, needs to be considered. Therefore, human reliability assessment of coal mine hoist systems is more complex and requires consideration of the state dependence of the hoist operator's cognitive behavior at multiple stages.

Many different analytical modeling strategies have been put out by numerous academics to address the issue of reliance and reliability assessment in PMSs. There are three main types.The first uses Monte Carlo simulation (MCS), a state-space-based approach, to construct state transfer models^10^. For instance, the phase dependence of each unit and the dynamic behavior of the system are represented by the Markov chain (MC) and Petri-net (PN) model^11^. The second utilizes combinatorial methods such as reliability block diagrams (RBDs)^12^, binary decision diagrams (BDDs), and multivalued decision diagrams (MDDs)^13^. Large-scale PMSs have been successfully handled by combinatorial methods based on decision diagram theory and Boolean algebra. The third and more applied one utilizes the classical probabilistic graphical model BN, a type of classical probabilistic graphical model that has gained popularity among researchers for its improved ability to describe probabilities and correlations in complicated systems^14,15^. An event-tree-based BN model, for instance, was proposed by Li et al.^16^ and can be used to assess the risk and reliability of PMS. Zhao and Smidts^17^ used Bayesian networks to represent human cognitive activities and Monte Carlo simulations to account for various uncertainties in cognitive processes. Human reliability study is aided by this cutting-edge environment for cognitive modeling and simulation. Xu et al.^18^ created a new reliability assessment method that assesses the dependability of multistate PMSs by using data gathered from various phases. A dynamic Bayesian network and a particular expectation maximization method were combined to achieve this. Overall, BN is a method for PMS reliability assessment that works well since it can accurately depict the dependencies across phases as well as the chance correlations inside a single phase.

In the past human reliability assessment studies, HRA has been widely used in various fields as a method to assess the probability of human error to improve the safety and reliability of systems.

A number of HRA techniques have been created in the past to assess and forecast human errors^19^. They analyze how human activity affects safety and reliability systems using systems engineering and cognitive and behavioral sciences, and they forecast the likelihood of human errors based on employee actions and decisions. In the study of human reliability in the field of coal mining, Wang et al.^20^ analyzed the behavioral reliability of traditional mechanized coal mining operators using Monte Carlo methods. The influence of sound in the working environment on the behavioral reliability of operators was explored, but the influence of human own factors on behavioral reliability was ignored. A fuzzy Bayesian network approach was used by Qiu et al.^21^ to create a gas tunnel safety risk assessment model. Liu et al.^22^ developed the Human Factors Analysis and Classification System for Coal Mines in China based on the statistical findings of 362 significant coal mine accidents that occurred in China. He also looked into the poor safety practices of coal mine workers and the factors that influenced those behaviors. Li et al.^23^ identified and evaluated the risk factors of coal mine safety production using text mining and Bayesian network technology, and they clarified the six main risk elements of coal mine safety production and their connected components. Fuzzy logic and Bayesian networks were coupled by Xue et al.^24^ to assess three fault states in the gas monitoring network. Fuzzy logic was used to represent the fault states of the system. The results of a thorough bibliometric analysis of the HRA field conducted by Hou et al.^25^ revealed that classic HRA approaches are inappropriate for usage in task and system transitions as well as the current workplace. To address these developments, new HRA models must be created. According to French et al.^26^, workers' cognitive processes as well as other performance-influencing aspects (safety culture, safety training, etc.) should be taken into account in order to further extend and improve HRA approaches.

In man–machine PMS, more attention has been paid to the interdependence between machine failures in the past, and less research has focused on the dependence of human cognitive errors. Also in the past human reliability studies have been more research focused on the dependence of human cognitive errors. Some researchers, for example, merely treat the operator as a machine unit without accounting for cognitive errors and the corresponding phase dependence in the operator's cognitive process, which is not representative of the actual scenario^27^. In genuine operational processes, PMSs also feature the phase dependence of human cognitive errors in addition to the phase dependence of machine states. Ignoring them will inevitably cause the evaluation results' accuracy to decline^11^. Most of the past human factors reliability assessments about the coal mining field have been focused on traditional coal mines, and there are still some shortcomings in human factors reliability studies for smart coal mines. In particular, it will be the focus of this paper to identify and quantify the performance impact factors of human factors reliability in human–computer interaction operations in smart coal mines.

In summary, this paper combines the operational characteristics of intelligent coal mine hoist systems, focusing on the cognitive behaviors and cognitive error dependencies of hoist operators during operation, and proposes a new human factors reliability assessment method combined with BN. Using Bayesian networks, the PSFs and related cognitive activities of hoist operators are considered as modeling elements, and the stage dependence of human cognitive errors are also well mapped into the BN, by which a human factor reliability model of the hoist system is constructed. Subsequently, the human factor reliability of the coal mine hoist system is evaluated based on the conditional state probabilities of all identified nodes. Finally, a typical intelligent coal mine is selected to evaluate the human factor reliability of coal mine hoist system. It enriches the research related to human factor reliability assessment in human–machine interaction system of smart coal mines and provides a theoretical basis for developing effective human factor failure prevention measures for smart coal mine hoist operations.

The rest of the paper is composed as follows: in "System descriptions", the stage dependence of human cognitive errors is analyzed, and the method and Bayesian network technique of how to determine the similarity of different operational stages are described. "Methodology" presents the human factors reliability assessment method and its steps in the hoist system proposed in this paper. "Case evaluation" carries out a typical example analysis. "Discussion" discusses the results of the analysis in "Case evaluation" and with previous studies. "Conclusion" gives the conclusions of this paper.

## System descriptions

### Phase dependencies analysis

In contrast to traditional single task systems, PMSs have phase dependencies between subsequent phases. Therefore, it is important to recognize and examine the phase dependencies of system units for modeling before evaluating the human reliability of coal mine hoist systems. Along with the phase dependency of machine states, phase dependence of human cognitive errors is a characteristic of PMSs. In the subject of HRA research^28^, the relationship between human-caused errors has come to be recognized as a phenomenon. The main stages of human cognition include, observation, comprehension, planning, and execution. Errors in observation, manipulation, and understanding in the previous stage affect the likelihood of the same errors occurring in subsequent stages when similar situations arise^29^. Therefore, they are analyzed comprehensively in this section.

**Figure 1:**
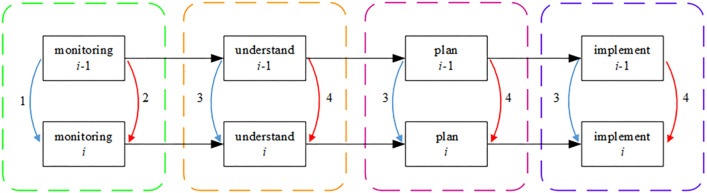
Shows the phase dependency of human cognitive mistakes. The red arrowed line denotes the facilitative effect of cognitive errors, whereas the blue arrowed line denotes the inhibitory effect. The arrow lines' numbers represent the many forms of dependency.

The reliance of cognitive errors between two neighboring phases can be categorized into four types, as shown in Fig. 1, depending on the chosen cognitive activity and cognitive phase. The hoist operator normally has to complete a series of interconnected cognitive tasks, although the task in phase *i* is affected by the task in phase *i − *1 (*i* = 2, 3, n, where n is the total number of phases).In terms of observation, this paper focuses mostly on gathering visual data through observation. As a result, two features, comprising (1) and, can be used to characterize the corresponding interdependencies (2). Furthermore, understanding, planning, and execution involve information processing based on the operator's cognitive-behavioral model and are therefore considered here as sharing similar phase dependence mechanisms. Therefore, the phase dependence of understanding, For instance, can also be characterized according to both aspects (3) and (4).

1. If the observation of *i − *1 phase is successful: this indicates that an object at phase *i − *1 was successfully detected by the operator. When a similar observation need arises in phase *i*, it is quite simple to capture the item again or even to forgo the observation^30^, lowering the possibility of errors (arrow line 1).
2. If the observation *i − *1 phase fails: without additional cues to remind of the previous error, the operator is more likely to make the same mistake again on observing a similar object in phase *i* (arrow line 2)^31^. Conversely, with additional reminders or cues, awareness of the previous phase error will increase one's attentiveness and can inhibit the recurrence of the same error (arrow line 1).
3. If the understanding of *i − *1 phase is successful: It indicates that the operator is using the appropriate cognitive model and is equipped with the relevant information or rules to manage the current circumstance. When a comparable circumstance arises in phase *i*, the operator may produce the right understanding once more (arrow line 3).
4. If understanding the *i − *1 phase fails; without further clues, having an incomplete cognitive model of the operator increases the likelihood that the scenario would be misunderstood again in the *i* phase (arrow line 4). The gap between the two phases is once again another crucial factor, even with extra cues. The operator will have the chance to complete his or her incomplete cognitive model if the time gap is reasonably long enough to enable correct understanding in the following phase (arrow line 3). Otherwise, under extreme time pressure, the operator will probably adopt a heuristic method and continue with trial and error^32^ (arrow line 4).

### Situational similarity analysis

**Figure 2:**
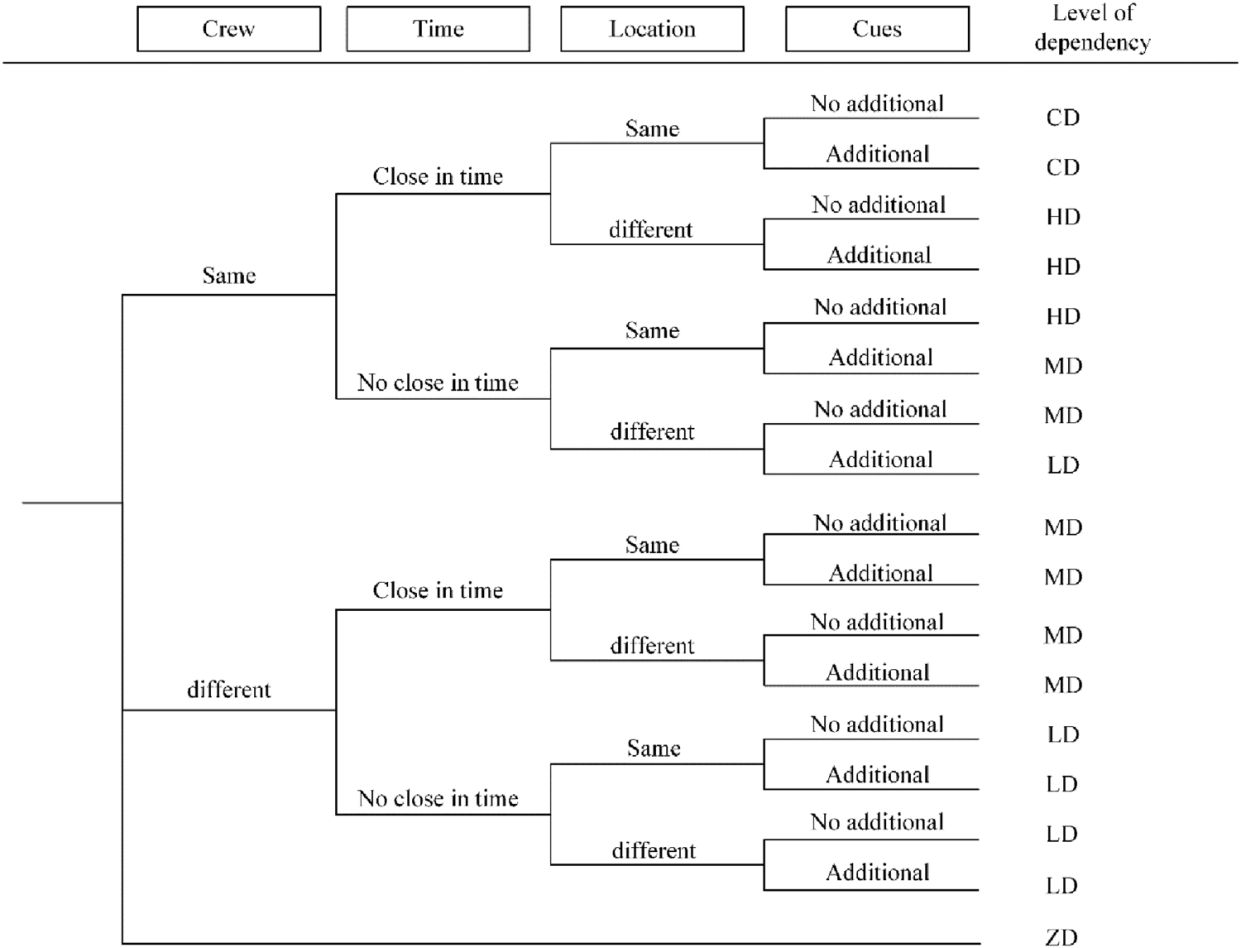
Decision tree for determining the degree of dependence of cognitive behavior.

The comparability of the scenarios between the two stages to enable the subsequent model generation and the subsequent estimation of the adjustment factor is a crucial prerequisite for evaluating the stage dependence of human cognitive mistakes. The decision tree (DT) suggested by Standardized plant analysis of risk-human reliability analysis (SPAR-H)^29^ is used in this research to provide a method for determining the degree of stage dependence of human cognitive errors. The decision tree (DT) proposed by SPAR-H is used in this research, as shown in Fig. 2.

It refers to the impact of an operator's failure to perform a subsequent task after failing to perform a previous task. The majority of HRA methods, notably SPAR-H, make the assumption that the probability of an operator failing or failing in the next task segment may be higher if the operator fails or fails in the task compared to when the operator succeeds. The key risk indicators may be greatly underestimated, and the ensuing results for human reliability may appear distorted, if the potential relationships between cognitive actions are not accurately portrayed. The intersection of each leaf node represents one of the four characteristics, including personnel, time, place, and cues, and each leaf node represents a level of assessed similarity. These levels are complete (CD), high (HD), moderate (MD), low (LD), and zero dependence (ZD). The impact on following cognitive processes increases with dependence level. The rules used to establish the various levels of reliance are shown on the path from the beginning to the finish in the DT. When two phases are more similar in terms of their position, their timing, and the presence of additional stimuli, there is a stronger degree of reliance between them. With regard to the aforementioned cognitive deficits in humans, the pathways in DT are compatible with and span a variety of phase dependencies. The similarity of the situation at each stage can be assessed in terms of five descriptive dependency levels. Subsequently, the independent HEP of the operator-related nodes in the later stages can be adjusted based on the assessed dependency levels. each dependency level corresponds to a specific adjustment factor α to adjust the HEP formula. Thus, the suggested modeling approach incorporates the DT for determining situation similarity.

### BN Model descriptions

BN are quantitative causal models consisting of a directed acyclic network and several statements about probabilities that characterize the dependencies between different PSFs and related activities. Nodes, arcs, and conditional probability tables(CPTs)are provided by BN^15^. These can be used to encode the many types of knowledge that operators keep in their memory, or what is known as the information processing model. In order to build a human reliability assessment model for hoist systems, BN was chosen as the modeling tool because it can accurately depict the causal linkages and dependencies between factors and targets in the PMSs of hoist systems.

The BN consists of two parts: the qualitative topology and the quantitative CPTs^33^. A directed acyclic graph made up of a collection of nodes V = {*X1, X2,⋯Xn*}connected by a collection of directed edges serves as a representation of the topology. The nodes represent the interesting random variables, while the directed edges define the causal or effect linkages between them. In this study's application of BN to express the causal relationship of flaws, the directed edge between two nodes serves as an example of the causative relationship. Instead of using probability intervals between them, a particular BN is used to translate expert expertise into human error probability (HEP) values for operators. Expert knowledge is transformed into HEP values for operators rather than probability intervals between them using a customized BN. If node *X*_*i*_ and node *X*_*j*_ are connected by a directed edge, *X*_*i*_ is referred to as *X*_*j*_ 's "parent" node and is represented by the symbol Pa. (*X*_*j*_). As a result, *X*_*j*_ is referred to as the "child" node of *X*_*i*_, signifying the causal relationship between Xi and. *X*_*j*_ A leaf node is a node without any children, whereas a root node is a node without any parents. By allocating unconditional distributions (which can be derived from statistics or domain experts) to root nodes and CPTs to intermediate nodes^34^. The joint probability distribution of the entire network can be written as follows using the chain rule of BN^35^.

1 $$P\left({X}_{1},{X}_{2},\cdots {X}_{n}\right)={\prod }_{i=1}^{n}P\left({X}_{i}\mid Pa\left({X}_{i}\right)\right)$$

where represents all BN nodes, and Pa(*X*_*i*_) denotes the parent set of node *X*_*i*_.

## Methodology

**Figure 3:**
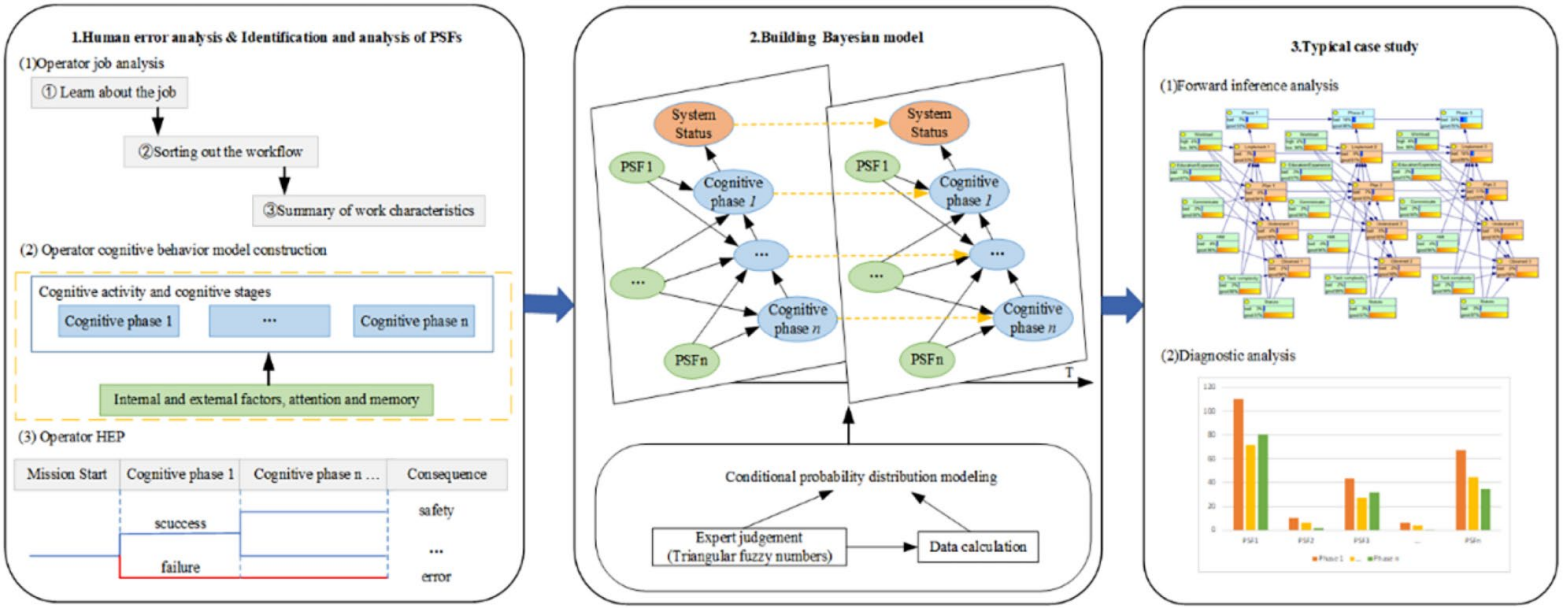
Research framework.

This study is broken down into three key steps as illustrated in Fig. 3 as follows. The PSFs in the hoist system are identified and screened in step 1 based on the cognitive behavior model that is created for hoist system operators. In step 2, with the aid of BN, an intelligent coal mine hoist system human factors reliability model is built, and the sources and processing techniques of various model parameters are introduced. Step 3 was completed to choose typical individual cases for validation and analysis in order to show the suggested method's viability.

### Cognitive model construction

According to traditional psychology, the human cognitive process consists of three main parts: first people perceive stimuli from external information, then the received information is transmitted to the brain for processing, and finally the response activity is made, which is the classical stimulus–organism–response (S–O–R) model^36^. Smidts et al.^37^ adapted the information processing model and proposed the (IDA) Information, Decision, Action model, and divided the cognitive process into four modules: information perception, decision, execution, and mental state. However, because it was too complex for practical application, it later evolved into the Skill-Rule-Knowledge (SRK) model, which classified human cognitive behavior into three behavioral modes: skill-based, rule-based, and knowledge-based^38^. In recent years, some scholars, divided the cognitive behaviors of operators in digital control rooms into four stages: monitoring, state evaluation, response plan, and operation, and established a model of operators' cognitive behaviors^39,40^, this may be useful for improving the relevant human reliability analysis model.

**Figure 4:**
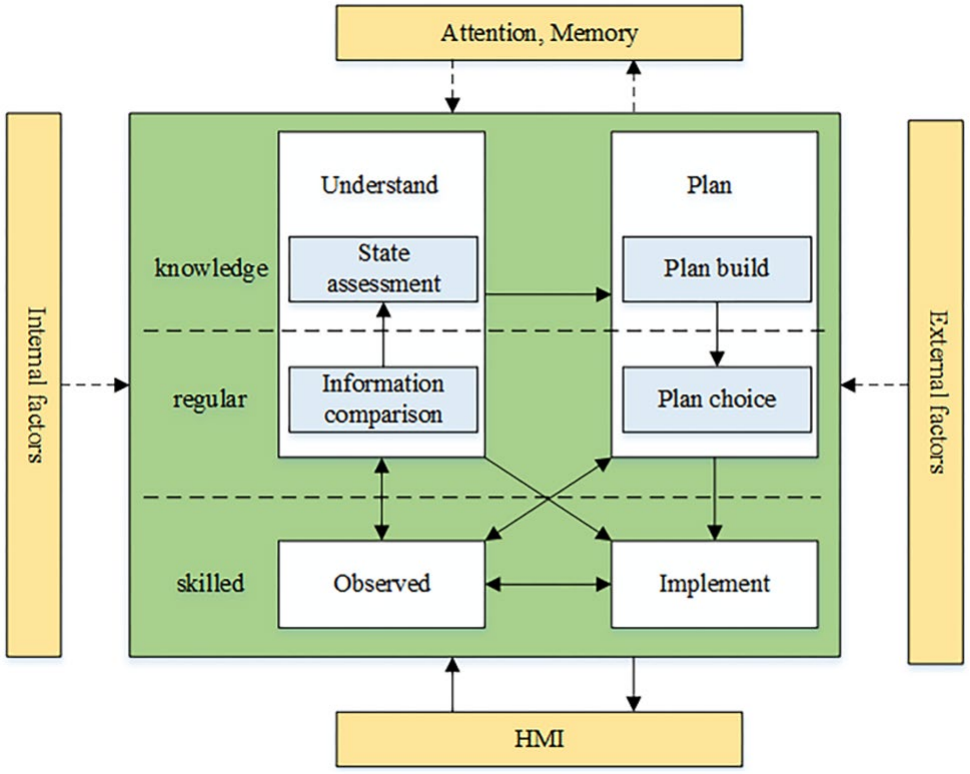
Cognitive behavior model of intelligent coal mine Hoist operator.

The cognitive behavior model, which is highly important in people's research on human mistake, examines the human cognitive process based on the analysis of various cognitive behaviors and cognitive functions of individuals. However, the previous cognitive behavior model is relatively simple, the interaction between the cognitive functions is not well described, and the influence of the external environment on cognition is not taken into account^41^, so it is difficult to fully reflect the dynamic cognitive behavior characteristics of human–machine interaction operation in intelligent coal mine hoisting machine room. Therefore, based on the analysis of human–machine interaction in intelligent coal mine hoisting machine room, considering the applicability of SRK model and the understanding of IDA model on the cyclic interaction process of cognitive functions and the relationship with mental state, this paper expands the SRK model on the basis of IDA model and adds internal and external factors that affect the cognitive process, and constructs the intelligent coal mine hoisting machine The driver's cognitive behavior model is shown in Fig. 4. The human cognitive behavior process is monitored and controlled by the Hoist operator through the human–machine interface. The operator can access the hoist parameter information through the display system of the HMI and assess the current status of the system. Then, based on this assessment, the state of the hoist can be determined and the operating procedure and path can be selected accordingly. Finally, control response tasks are performed. Thus, the cognitive-behavioral process of a coal mine Hoist operator is characterized by four phases: observation, understanding, planning and execution.

The whole cognitive process constructed by the model is driven by mental factors such as attention and memory, while each cognitive function will change the state of mental factors in real time during the execution process, and the cognitive function and mental factors are dynamic interaction process, and the cognitive process will also be influenced by the Hoist operator himself and the surrounding environment. In summary, the cognitive behavior model emphasizes the interaction between cognitive functions and mental states, the non-serialization of cognitive functions and the role of internal and external factors on the cognitive process.

### Analysis of human error in hoist systems

Human errors are simply caused by people's incorrect cognitive behavior. The cognitive processes of hoist operators and the completed work analysis were examined in the previous paper. The next stage is to investigate what leads to hoist operators' incorrect cognitive activity and sets off human errors. Human errors will also be generated by the situational environment in which the person is positioned, it is currently generally accepted that attributing human errors solely to the person's own causes is biased.

Factors that contribute to human error include at least those related to the person, task-related, man–machine interface, environment, and interactions between people in the organization. Through the study of existing behavior formation factor classification methods, such as 67 PSFs provided by THERP, 9 CPCs by CREAM, 8 PSFs by SPAR-H^42,43^ and others, the behavior formation factors that may lead to human-caused errors are summarized by combining the characteristics of man–machine interaction operations of intelligent coal mine hoists, as shown in Table 1.

**Table 1:** Intelligent coal mine Hoist operator behavior formation factors.

Category	Subcategories	Specific factors
Personal	Physiological status	Fatigue
	Mental state	Stress; attitude
	Qualities and competencies	Attention; knowledge; experience; memory
Mission	Mission characteristics	Task complexity; available time; task novelty; workload
	Statute	Usability; completeness; comprehensibility; level of detail
Human machine interface	Panel/screen	Ease of information identification; layout rationalization
	Hardware	Equipment reliability; inspection and maintenance
Environment	Working environment	Noise
Organization	Safety culture	Safety regulations; security awareness
	Education and training	Skills training; safety culture training
	Communicate	Information communication and feedback
	Management issues	Inadequate management
	Organizational procedures	Rationalization of work procedures

The factors influencing different cognitive functions can vary, and the role of different behavioral formative factors in influencing different cognitive functions varies. Therefore, it can be assumed that different behavioral formation factors lead to different types of person-factor failures, i.e., behavioral formation factors and person-factor failure patterns are closely related. Based on the identification of the factors influencing cognitive functions, the relationship between human error patterns and behavioral formation factors was analyzed, as shown in Table 2.

**Table 2:** Relationship between human error patterns and behavior formation factors.

Serial number	Human error mode	Behavior forming factors
E1	Not observed/discovered	Fatigue; attention; equipment reliability; noise; safety awareness
E2	Observation error	Fatigue; attention; layout rationality; ease of information identification
E3	Misidentification	Knowledge; experience; ease of information identification; skills training
E4	Diagnostic errors	Stress; knowledge; experience; memory; skills training
E5	Inappropriate planning	Stress; knowledge; experience; task complexity; available time; task novelty; information communication and feedback
E6	Wrong choice	Knowledge; experience; task complexity
E7	Action omission	Stress; attention; memory; task complexity; available time
E8	Action error	Stress; attitude; safety regulations; safety awareness
E9	Information is not exchanged	Information communication and feedback
E10	Incorrect/incomplete information exchange	Information communication and feedback

### Cognitive model-based operator PSF identification and analysis

Usually, as comprehensive as possible behavior formation factors are needed for humans failure identification, because only then can the causes of humans failures occur be identified more accurately, while such comprehensive behavior formation factors, which must be included at every level, are not needed for the calculation of humans failure probability. Among the commonly used human reliability analysis methods, SLM chooses six PSFs, CREAM uses nine CPCs, and HCR, HDT, and other methods choose behavior formation factors that are basically around a dozen. The reason why a small number of important behavior formation factors are usually used for human error probability calculation is mainly because when conducting quantitative analysis of human reliability, quantitative evaluation of each behavior formation factor is required, and the process of quantitative evaluation is complicated, and once the number of behavior formation factors is too many, it is likely to have the possibility of double calculation, which makes the results inaccurate instead. Interviews with seven experts were therefore conducted based on the identification of hoist operator PSFs. These experts included two university experts who had studied coal mine reliability for a long time, two hoist operators who had worked for more than 7 years, and two coal mine accident investigation experts who were primarily in charge of accident scene investigation, identification of accident elements, and determination of accident nature and direct cause. Among the PSFs in Table 2, they were asked to identify the primary PSFs for hoist operators in the four stages of observation, comprehension, planning, and execution. Final screening revealed six most prevalent PSFs, each of which had two states as indicated in Table 3, as markers for the quantitative examination of human reliability of hoist operators.

**Table 3:** Intelligent coal mine Hoist operator human reliability behavior formation factors.

Serial number	PSF	Description
PSF1	Statute	Usability; completeness; comprehensibility; level of detail
PSF2	Task complexity	Type of task; repetitiveness of tasks; number of parallel goals/tasks; task requirements
PSF3	Human machine interface	Layout rationality; information communication and feedback; difficulty of operation
PSF4	Communicate	Information communication and feedback
PSF5	Education/experience	Knowledge; experience
PSF6	workload	Physical load; mental load; mental load

### Reliability analysis

A BN-based technique for evaluating the human factor dependability of intelligent coal mine hoist systems is developed, which takes into account the stage dependency of human cognitive errors and the key human factor reliability PSFs of such systems in the following three steps.

#### Qualitative modeling construction

The BN model qualitative structure must first be constructed using the identified nodes as a base. The BN structure can change between phases to reflect how the PMSs are configured dynamically. The following three sub-steps can be added as further divisions of this step.

**Figure 5:**
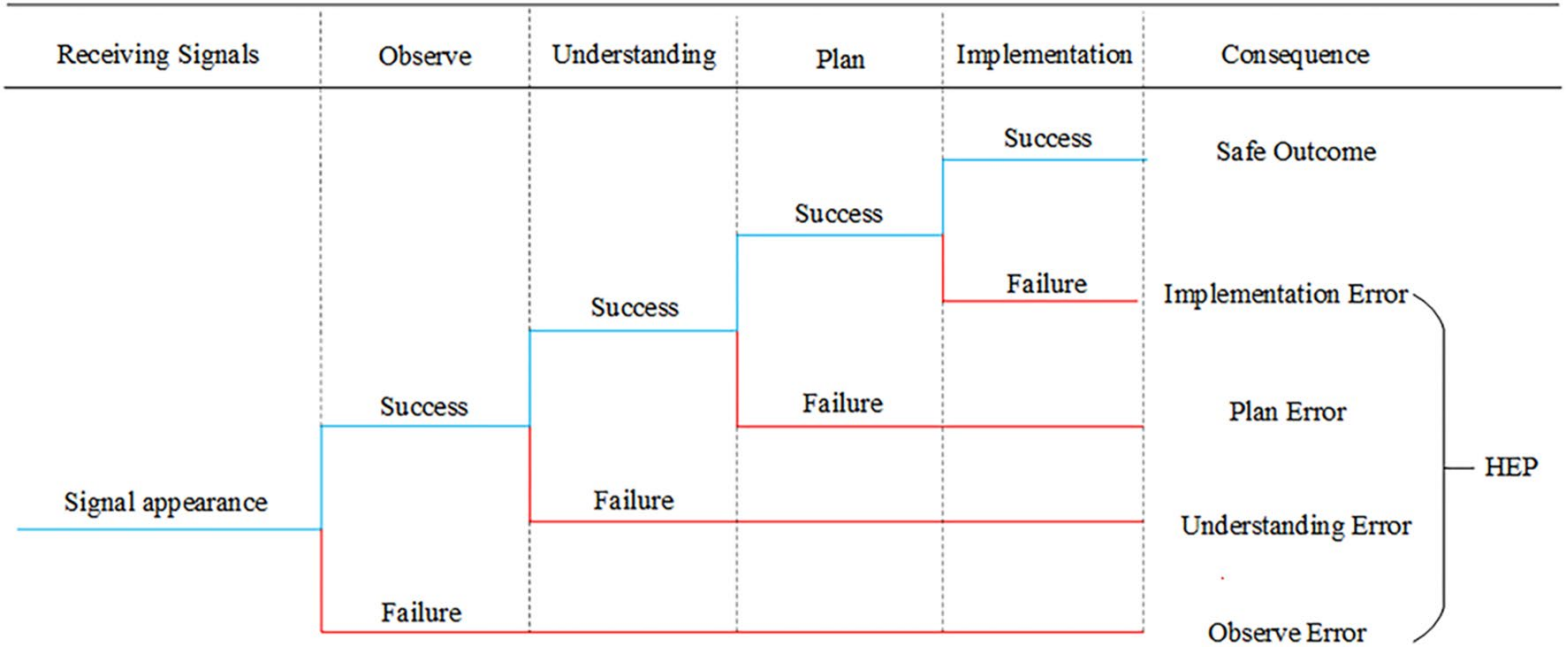
Event tree representation of the hoist operator HPE.

1. Ascertain each network node status. The determination of many requisite states for nodes that play a role may be necessary to help further quantitative modeling. Finding descriptive states (such as failure or success) for a specific node that is performing well or not functioning is one method advised for describing states. Degradation-like intermediate states can also be found, if necessary. To describe the operator-related nodes, this study, however, only takes into account two states.
2. Identify the nodes' causal connections within each phase. To create a qualitative BN model for a specific phase, causal linkages between various nodes should be established. Causative development begins at the root node and travels along arcs to the intermediate and target nodes. The BN that was created can be viewed as an illustration of the cognitive-behavioral framework that the operator uses to comprehend the current circumstance. The HRA event tree can be used to determine the causal connections between operator-related nodes1^44^. According to Fig. 5 HRA event tree, the final operation is only effective if all earlier cognitive tasks were completed correctly, in the proper order, and without any mistakes made by humans.
3. Establish connections between various phases. In order to develop an integrated model, this sub-step introduces various phase dependencies, which connect the established BNs of each phase. Linking the identical nodes of all phases in a chronological order is one method for illustrating the phase interdependence of the unit-level nodes (including the cognitive behavior of the hoist operator). Additional binary nodes, also known as indicator nodes, are added to demonstrate whether the hoist operator is functioning properly at the conclusion of each phase in order to prevent too many state combinations of CPT generated by directly connected target nodes. It is simple to describe the logical relationship between the human reliability of the hoist operator completing all phases by connecting the indicator node with a directed arc between each pair of neighboring phases.

**Figure 6:**
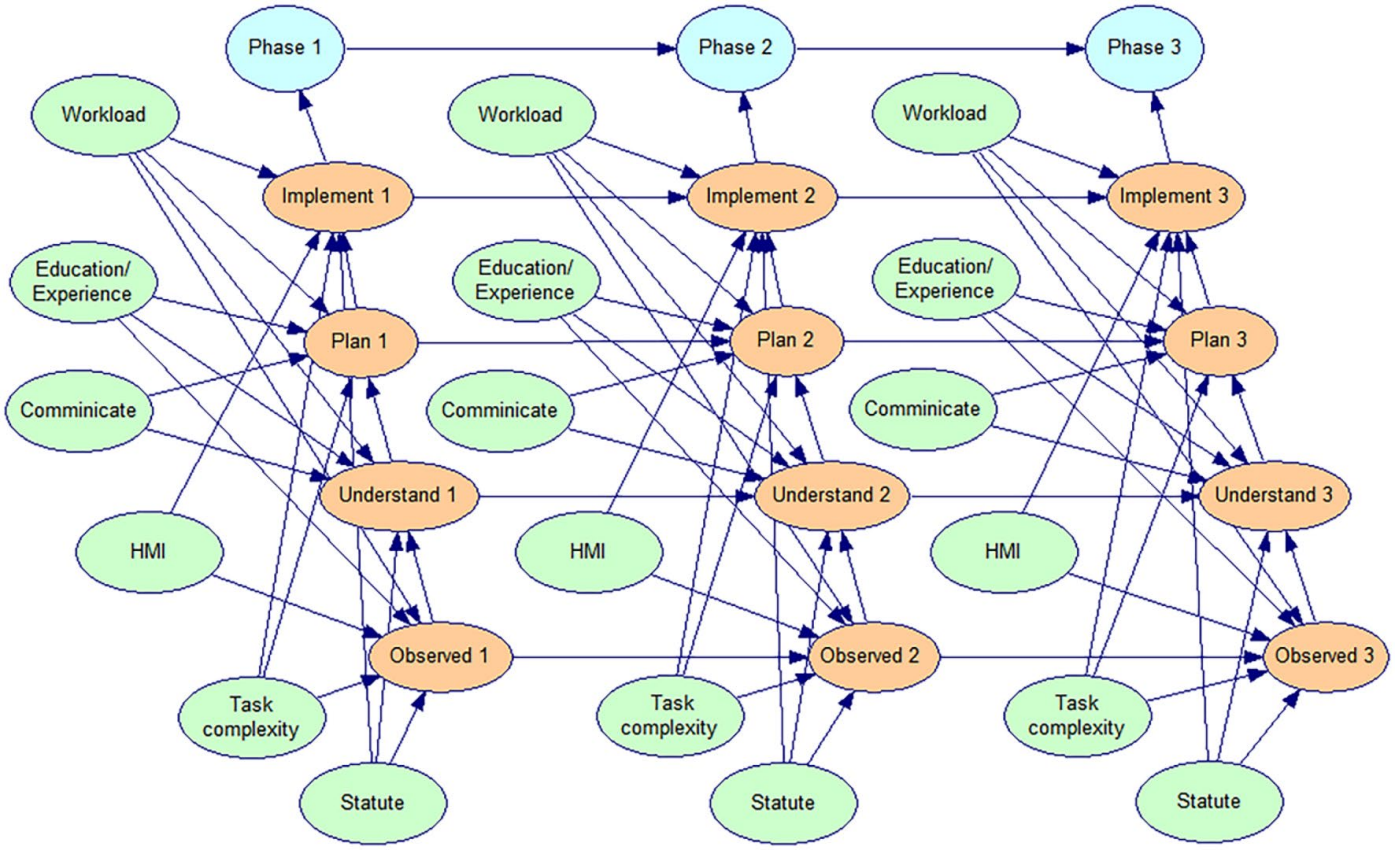
Qualitative structure of the BN model for human reliability of coal mine Hoist operators.

The three phases of lift man, lift object, and lift explosive, which correlate to phases 1, 2, and 3, are called for the target nodes for each phase. The root node represents the underlying cognitive behavioral influences, such as "Statute" and "HMI"; each phase of cognitive behavior is set as an intermediate node. Then, by linking the corresponding nodes, the phase dependencies in the human reliability system of the hoist operator can be constructed. In order to create a comprehensive qualitative intelligent coal mine hoist system human reliability BN model, the causal relationship between each phase's nodes is integrated with the phase dependencies of the nodes between various outcomes, as shown in Fig. 6.

#### Quantitative modeling

##### Calculate HEPs within each phase

In the Bayesian network model, ascertain the prior probabilities of the root nodes and the conditional probabilities of the intermediate and leaf nodes. The probabilities can be determined in a number of different ways, such as by identifying the connections between the nodes and constructing functional relations from data from experiments or simulators, by obtaining the probabilities under various conditions through mathematical analysis of a sizable amount of previously gathered data on human-caused error, or by consulting relevant literature, manuals, or domain experts. According to this approach, all associated cognitive activities are classified as diagnostic tasks and are consequently given the NHEPs that correspond to each phase. Second, in order to evaluate the existing working conditions, PSFs must be graded for each phase. Workload flexibility, complexity, experience/training, procedures, ergonomics/human–machine interface (HMI), stress/stressful stimuli, and available time are the eight PSFs that SPAR-H offers. Each PSF has three to five rating levels and corresponding multiplier values. It is feasible to quantify the phase dependency of the node by changing the CPT in the later phases based on the CPT in the earlier phases, which is selected by domain experts based on the definition and level of the PSF employed in SPAR-H. For each phase, the remaining PSFs can be suitably set to the same value. By multiplying the associated NHEPs by the PSFs, it is possible to determine the independent HEPs of the model-related nodes. The HEP adjustment algorithm is used when many PSFs are evaluated negatively:

2 $$HE{P}_{{x}_{i}}=\frac{NHE{P}_{{x}_{i}}\times PS{F}_{{C}_{i}}}{NHE{P}_{{x}_{i}}\times \left(PS{F}_{{C}_{i}}-1\right)+1}$$

where *HEPxi* and *NHEPxi* refer to node x's independent HEP and NHEP, respectively, in the first phase.

##### Quantifying the phase dependence of human cognitive error

In this sub-step, a further adjustment of the independent HEP is necessary to quantify the effect of the phase dependence of human cognitive errors. First, for each sort of cognitive activity, it is important to assess how closely the two adjacent phases resemble each other as they might not involve exactly the same circumstances. The comparability of events can be evaluated in terms of five descriptive dependency levels by utilizing the DT depicted in Fig. 2. In the following phases, the operator-related nodes' independent HEPs can be modified using the determined dependence levels. Here, the dependencies are quantified using the SPAR-H method for generating conditional HEPs^45^. In the event that the identical action from the prior phase fails, each dependency level corresponds to a particular variation of the HEP formula:

3 $$HE{P}_{{x}_{i}\mid XD}=\frac{1+\alpha \cdot HE{P}_{{x}_{i}}}{\alpha +1},XD=ZD,LD,MD,HD,CD$$

Here, the adjustment factor α and the adjusted HEP at the dependent level of XD are displayed. Table 4 displays the numbers. The parameter values are drawn from the most often used quantitative correlation model provided by the basic HRA approach, commonly known as the human error rate prediction(THERP) technique. Describe how cognitive mistakes facilitate. Calculating the nodal likelihood of success under the presumption that the identical activity was successful in the previous phase will yield similar findings (the inhibitory effect of cognitive errors)^45^:

4 $$HS{P}_{{x}_{i}\mid XD}=\frac{1+\alpha \cdot HS{P}_{{x}_{i}}}{\alpha +XD},ZD=ZD,LD,MD,HD,CD$$

where $HS{P}_{{x}_{i}\mid XD}$ stands for the node's success likelihood at the XD dependent level.

**Table 4:** Value of adjustment factor α.

Adjustment factor	CD	HD	MD	LD	ZD
$a$	0	1	6	19	∞

The logical relationship between the associated nodes in the BN model can also be used to calculate the CPT of the hoist system's human reliability-related nodes. When the individual PSF nodes have been identified, the basic reasoning behind the CPT assignment of the intermediate relevant nodes can be stated as follows: if the hoist operator's prior cognitive activity in the current phase failed or was performed incorrectly, the state of that cognitive activity node is incorrect; alternatively, its state probability is conditionally decided based on the condition of the same kind of cognitive activity in the previous phase. For instance, if phase *i − 1* and phase *i* depend on MD, Table 5 displays the CPT of the node plan_*i*_ in phase *i*. The same method can be used to determine the CPT of additional nodes.

**Table 5:** The CPT of the node plan_*i*_ in the phase *i.*

PSF	Bad/Good			
Understand_*i*_	Bad		Good	
Plan_*i-1*_	Bad	Good	Bad	Good
Bad	1	1	$HE{P}_{{x}_{i}\mid XD}$	1 − $HS{P}_{{x}_{i}\mid XD}$
Good	0	0	1 − $HE{P}_{{x}_{i}\mid XD}$	$HS{P}_{{x}_{i}\mid XD}$

#### System reliability assessment

The chain rule of CPT and BN of all identified nodes can be used to determine the joint probability distribution of the target nodes in each phase of this phase. It goes without saying that the task state of the entire human–machine system at this phases is only successful if both the operator's (*RPi*) cognitive activity and the machine system's operation are successful. Following that, the conditional probabilities listed below can be used to describe the logical relationship between the task success criteria at each phase:

5 $$P\left(I{N}_{i}=1\mid I{N}_{i-1},T{N}_{i}\right)=\left\{\begin{array}{cc}1& I{N}_{i-1}=1,T{N}_{i}=1\\ 0& \text{ else}\end{array}\right.$$

where *IN* and *TN* stand for, respectively, the indication node and the target node (the condition of the system during the current phase). Thus, the probability of success of the indicated node in the final stage reflects the probability that the operator will maintain proper operation without human error throughout the phased-mission of the intelligent coal mine hoist system.

## Case evaluation

### Get the prior probability of the root node

For the determination of the a priori probability and conditional probability of human reliability PSFs of intelligent coal mine hoist operators, they cannot be derived by mathematical analysis because of the absence of a large amount of human-caused failure data. The assessment of the a priori probability and conditional probability of PSFs has been done, and the nuclear power plant operators' a priori probability and conditional probability of PSFs have been addressed with triangular fuzzy numbers and appraised using the expert interview method^46^. However, given that the coal mine hoist room environment is somewhat different from theirs, it is not possible to use their data directly, and there is a lack of basic data on intelligent coal mine hoist rooms to obtain the probabilities of certain situational environmental states. Therefore, this paper uses expert scoring method to determine the prior probability and conditional probability of each node.

**Figure 7:**
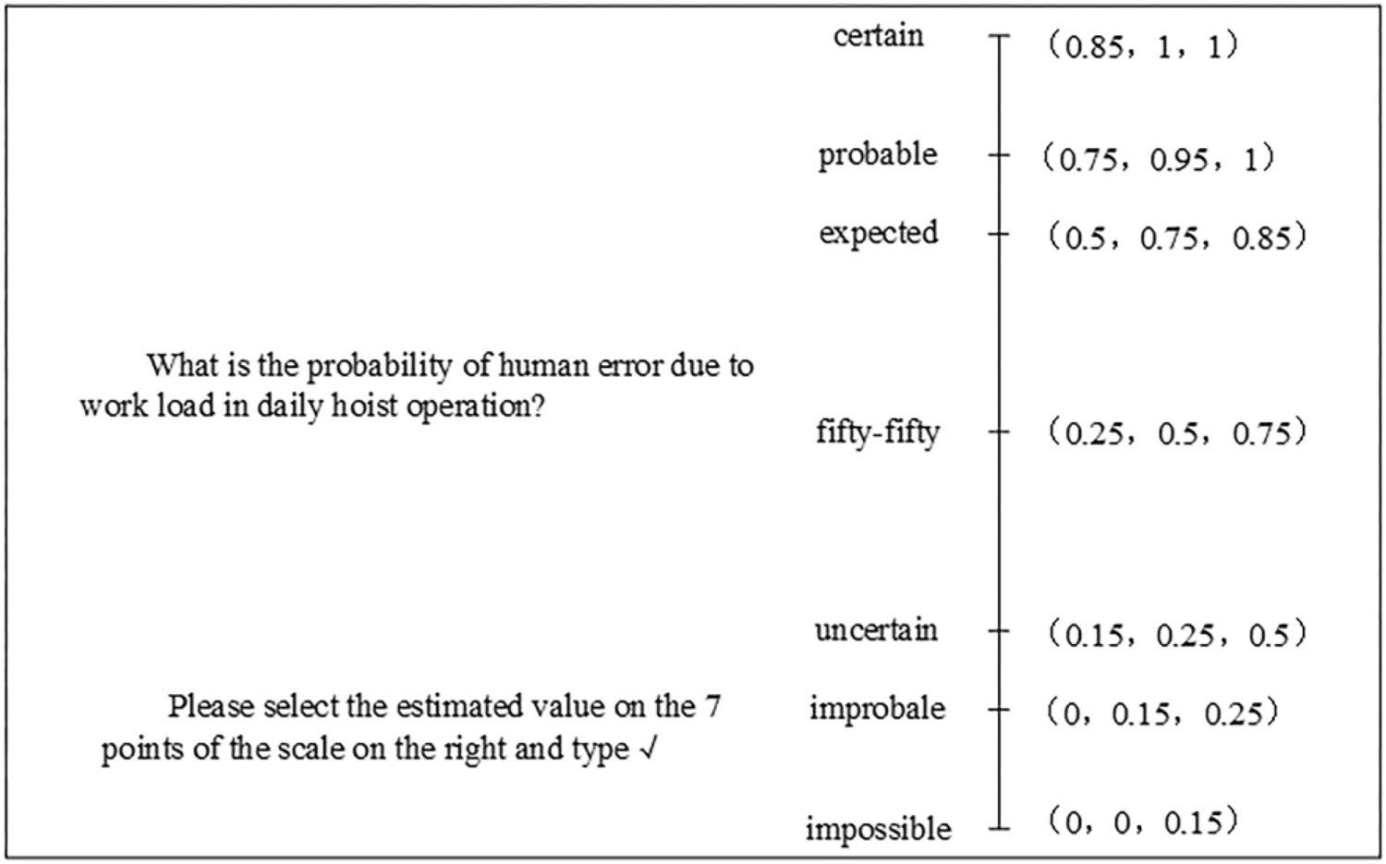
Qualitative structure of the BN model for human reliability of coal mine Hoist operators.

One intelligent coal mine humans expert, two more intelligent coal mine hoist operators and one safety inspector, a total of four experts, were selected for scoring and allowed to evaluate the prior probability of the root node, the conditional probability of the intermediate node and the leaf node. To reduce the influence of subjectivity in the evaluation, a triangular fuzzy number estimation range is given in the evaluation, which is divided into seven grades, and the probability of each node being in different states under different scenarios is selected. Then a fuzzy number semantic-numerical probability scale is designed accordingly and a semantic questionnaire is developed (as shown in Fig. 7) to facilitate experts, hoist operators and safety inspectors to evaluate and score the probabilities under different scenarios.

Since the data given by experts, Hoist operators and safety inspectors differ in their working experience and professional knowledge, the collected data cannot be simply averaged, and the obtained triangular fuzzy numbers need to be averaged, defuzzified and normalized to finally obtain a priori probabilities and conditional probabilities that match the actual situation.

6 $$ \varphi ({\text{x}}) = \left\{ \begin{gathered} 0,x < m; \hfill \\ (x - m)/(b - m),m \le x < b; \hfill \\ (n - x)/(n - b),b \le x < n \hfill \\ 0,x \ge n \hfill \\ \end{gathered} \right. $$

The triangular fuzzy number affiliation function is shown in equation.

The triangular fuzzy number probabilities are first averaged. The probabilities given by different experts are arithmetically averaged, then the average of the probabilities of the nodes is:

7 $${\widetilde{P}}_{ij}^{\mathrm{^{\prime}}}=\frac{{\widetilde{P}}_{ij}^{1}+{\widetilde{P}}_{ij}^{2}+\dots \dots {\widetilde{P}}_{ij}^{l}}{l}=({m}_{ij}^{\mathrm{^{\prime}}},{b}_{ij}^{\mathrm{^{\prime}}},{n}_{ij}^{\mathrm{^{\prime}}})$$

Finally, the probability values are normalized so that the sum of the probability values of each node in different states is 1. The final probability values are then obtained.

8 $${P}_{ij}^{^{\prime}}=\frac{{\widetilde{P}}_{ij}^{^{\prime}}}{{\sum }_{j=1}^{k}{\widetilde{P}}_{ij}^{^{\prime}}}$$

By processing the obtained data, the prior probabilities of the six root nodes were obtained, as shown in Table 6.

**Table 6:** A priori probabilities of root nodes affecting the reliability of Hoist operators.

Node	Status and probability	
Statute	Good 0.98	Bad 0.02
Task complexity	Complex 0.97	Simple 0.03
HMI	Good 0.96	Bad 0.04
Communicate	Adequate 0.98	Inadequate 0.02
Education/experience	Good 0.97	Bad 0.03
Workload	High 0.96	Low 0.04

### CPT computation method

The CPT assignment procedure of the node Observed2 can be described as follows. First, the NHEP is set to 0.01 because the observed error mode is "observation error". Second, based on the SPAR-H scoring table to produce scoring Table 7, according to the PSF level in phase 1, assuming a moderately complex task, normal protocols, poor human–machine interface, normal communicate, normal education and training, and normal workload. The composite PSF can be calculated as *PSFs* = 2 × 1 × 0.5 × 10 × 1 × 1 = 10. The independent HEP for Observed1 can be calculated by multiplying the NHEP by the composite PSF:$HEP=\frac{0.01\times 10}{0.01\times (10-1)+1}=0.0917$. Fourth, the independent HEP can be changed to the condition using the updated formula for HD depending on the assessed level of dependence (HD): *HEP* = (1 + 0.0917)/2 = 0.54585, *HSP* = [1 + (1 − 0.0917)]/2 = 0.95415. conditional probabilities for other cases at the PSF level can be obtained in a similar way. Finally, the values of HEP and HSP obtained for various cases are brought into Table 5, and the CPT of each node can be obtained, which is input into GeNIe software for analysis.

**Table 7:** PSFS and their multiplier for the diagnosis and action portion of the task.

PSF	Selected level	Multiplier
Task complexity	Highly complex	5.0
	Moderately complex	2.0
	Nominal	1.0
	Obvious diagnosis	0.1
Statute	Not available	50.0
	Incomplete	10.0
	Available, but poor	5.0
	Nominal	1.0
	Diagnostic/symptom oriented	0.5
HMI	Missing/misleading	50.0
	Poor	10.0
	Nominal	1.0
	Good	0.5
Communicate	Poor	2.0
	Nominal	1.0
	Good	0.8
Education/experience	Low	10.0
	Nominal	1.0
	Good	0.5
Workload	Extreme	5.0
	High	2.0
	Nominal	1.0

### Model runs and data analysis

#### Face validity

Regarding the system's human reliability logic, the evaluation of face validity focuses on determining whether the calculation of each node, network structure, and model conditional probability is comparable to the experts' empirical knowledge. It also examines whether it accurately depicts the real hoist operation situation. Four more experts were recruited to take part in the validation and assessment of the suggested method because it was difficult for the four experts participating in determining the model a priori probability to disagree with one another. Two of the four specialists were hoist operators with expertise, while the other two were scholars working on studies related to coal mine safety management. The four experts concurred that the nodes and their connections largely matched their empirical understanding and the results of relevant research. Additionally, the model fulfills face validity since the probability distributions of the nodes and the model's parameters somewhat mimic the functionality of the real equipment.

#### Forward inference analysis

**Figure 8:**
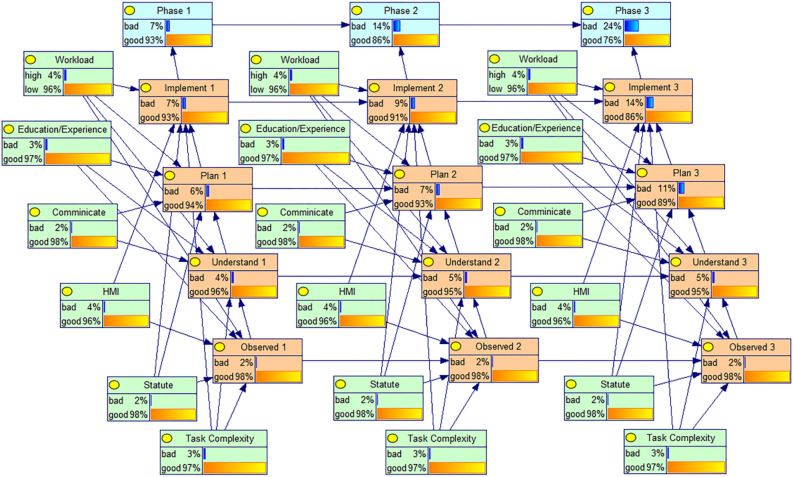
Quantitative assessment model of human reliability of coal mine Hoist operators.

By entering evidence at its parent nodes using the formula, the forward inference analysis based on the BN model can compute the probability distribution of the integrated processor system's failure lifetime. Once all nodes' conditional probabilities are known, it is possible to estimate their state distribution in any phase using the computation procedure described above. It is also possible to assess the hoist operator's dependability during each phases. The reliability of the Hoist operators in each phase can also be evaluated. The obtained prior probabilities and conditional probabilities are fed into the already constructed Bayesian network model of human reliability through the GeNIe software. Given the prior probabilities, the reliability and humans failure probabilities of each phase of the cognitive behavior of the Hoist operator can be derived, as shown in Fig. 8.

It can be observed that the reliability of the Hoist operator gradually decreases as the task progresses. These happen as a result of the system's design and the rising HEP levels between stages. The dynamic PSFs levels can be used to explain the variations in human reliability changes in the three phases. For instance, the operator's cumulative effects of fatigue were felt in phase 3 as opposed to phase 2 due to the lengthy working hours, which caused a comparable rise in PSF levels and a more abrupt decline in system reliability. Also, the reliability of each cognitive phase decreases more from phase 2 to phase 3 than from phase 1 to phase 2. In order to compare the variability of reliability across cognitive phases, the reliability in each of the three task phases is shown in Table 8. Unsurprisingly, the reliability of the Hoist operator gradually decreased in the four cognitive phases Observed, Understand, Plan, and Lmplement, and since the prior probability of each PSFs node set in each phase is the same, this indicates that the dependence of cognitive errors has a negative impact on the reliability of the Hoist operator.

**Table 8:** Human reliability of Cognitive Phases.

Cognitive phase	Phase 1	Phase 2	Phase 3
Observed	0.97574	0.97561	0.97557
Understand	0.95793	0.95162	0.94848
Plan	0.94452	0.93080	0.88999
Implement	0.92742	0.90667	0.86071

In the observation phase of the Hoist operator's probability of error is the lowest, the highest reliability, which is related to the advancement of coal mine intelligence, the hoist room monitoring and control devices are also gradually upgraded, man–machine interaction is more friendly, sound and light signals, functional partition layout, color highlighting, etc. can make the Hoist operator quickly find the problem, thus reducing the Hoist operator's errors in the observation phase. The execution phase has the highest probability of errors. With the increasing reliability and automation of the equipment, the cognitive tasks of the Hoist operator are increasing, and in the face of a large amount of information and audio and visual signals on the screen display, after perceiving and understanding the information, he needs to make a quick judgment, make a plan and carry out a series of operations, which may cause excessive pressure on the Hoist operator and make execution errors easily.

#### Diagnostic analysis

**Figure 9:**
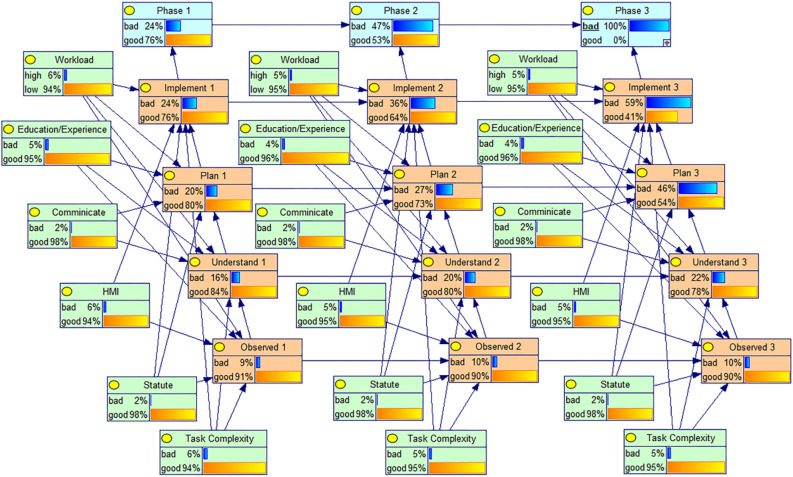
Coal mine Hoist operator human reliability diagnosis model.

The BN model was used to identify the root causes of failures that were caused by people. The BN established in the previous section is then evaluated based on the results of the fault diagnosis by doing a sensitivity analysis to examine the impact of changes in probability in the parent node on the child nodes. Contrary to causal reasoning, diagnostic reasoning examines the source of the outcome when the outcome is already known. In the BN model for human reliability analysis, assuming that a human failure has already occurred, one goes along the already built BN model to reverse the diagnostic reasoning to get the posterior probability of the root node and compare it with the prior probability to get the percentage change in probability, which shows which factors have a greater impact on human reliability. Therefore, assuming that a failure occurs in the lifting explosive phase, the posterior probability of its root node is reasoned and the diagnostic reasoning is shown in Fig. 9.

The posterior probability of each PFSs reliability root node is calculated by running it in the software as shown in the figure. Comparing them with the previously obtained prior probabilities, the percentage change of the posterior probabilities with respect to the prior probabilities can be obtained, as shown in Table 9.

**Table 9:** Percentage change of posterior probability with respect to prior probability.

Phase	PSF1	PSF2	PSF3	PSF4	PSF5	PSF6
**Phase 1**						
Prior probabilities	0.03	0.02	0.04	0.02	0.03	0.04
Posterior probabilities	0.0630	0.0221	0.0574	0.0213	0.0502	0.0566
Growth rate(%)	110	10.5	43.5	6.5	67.333	41.5
**Phase 2**						
Prior probabilities	0.03	0.02	0.04	0.02	0.03	0.04
Posterior probabilities	0.0515	0.0213	0.0510	0.0208	0.0433	0.0509
Growth rate(%)	71.667	6.5	27.5	4	44.333	27.25
**Phase 3**						
Prior probabilities	0.03	0.02	0.04	0.02	0.03	0.04
Posterior probabilities	0.0541	0.0204	0.0527	0.0201	0.0404	0.0479
Growth rate(%)	80.333	2	31.75	0.5	34.667	19.75

**Figure 10:**
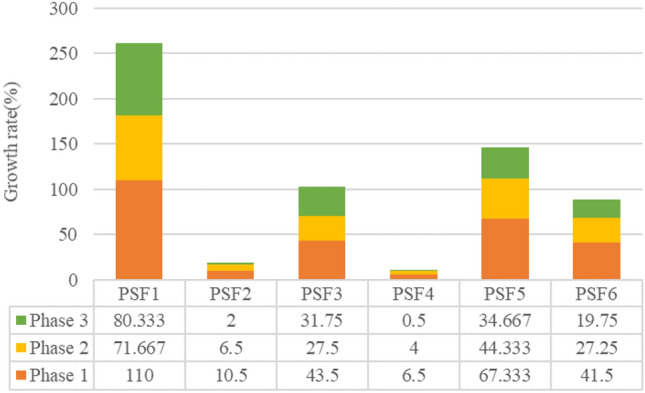
Superposition of posterior probability and percentage change of prior probability.

In order to show more visually the degree of influence of the root node of each PSFs on the human reliability, the posterior probability increase of the root node of these PSFs was plotted as a bar chart, as shown in Fig. 10.

Through the Table 8 and Fig. 10, it can be found that when the human reliability of the third-phase hoist system is 0, the PSF1 task complexity, PSF3HMI, PSF5 education/training, and PSF6 Workload posterior probabilities change more compared to the a priori probabilities, where the sum of the posterior probability increases of Task complexity, HMI, and education/training in the three-phase task are more than 100%, and Task complexity is more than 260%. This indicates that Task complexity has the greatest impact on the human reliability of the coal mine hoist system, followed by education/training, HMI, and workload, respectively, and these PSFs also have a significant impact on the human reliability of the hoist system. The posterior probability of PSFs in the diagnostic inference analysis was greater in phase 1 compared to the increase, which indicates that phase dependence still exists in the reverse diagnostic inference process.

The above facts prove that the interdependence of cognitive behaviors and human cognitive behaviors have a significant impact on the reliability of coal mine hoist operators. To address the problems of complex operational tasks, inadequate education and training, and high workload, the workflow of hoist operators should be optimized to reduce the complexity of their operational tasks and to improve the usability, operability, information recognition, and complexity of interface management tasks of the human–machine interface to reduce the workload of hoist operators. The operator's cognitive-behavioral training should also be improved, and the operation method should be standardized to aid in the operator's observation, understand, plan and execute correctly, and ensure the reliability of task completion.

## Discussion

The development of smart coal mines has led to the digitization of hoist systems and the diversification of operator operational tasks, and in the face of the human reliability assessment problem in smart coal mine systems, most of the existing human reliability assessment methods are used in conventional coal mines and rarely manage to consider the stage dependence of human cognitive errors and how they affect their own safety performance. Therefore, the appropriateness of traditional human factors reliability assessment methods needs to be further explored. This paper is a preliminary study of human-caused reliability assessment in smart coal mines, and discusses the proposed methods and limitations of human reliability assessment in smart coal mines considering the interdependence of human cognitive errors.

First, in our proposed approach, since the human factors reliability model for hoist operators is only used to quantify the HEP of operator-related nodes, it cannot change the conclusion that the interdependence of cognitive errors affects system reliability. However, the error-generating factors (which can be described by PSFs) considered in different HRA methods are more or less different, and this should also be considered when modeling coal mine hoist operators, therefore, the PSFs in the smart coal mine hoist system were identified and analyzed, and finally screened, which is more compared with the PSFS selection process in other human-causal reliability studies fit the actual situation of smart coal mine production, but the screening method relies only on expert judgment is more subjective and objective data analysis is not sufficient, and future work will make up for this.

Secondly, in the past, the reliability research about coal mine system has focused more on the external environment and equipment on the reliability of the system. However, in the context of intelligent coal mines, the reliability of equipment is increasing and the external environment is simpler and safer, so it is more important to focus on the reliability of people in the system. In this paper, we focus on the reliability assessment of human in the system, and the results obtained are slightly different from the past studies. Past studies have concluded that environmental factors^20,47^, human emotions, and some physiological abnormalities^48^ have a greater impact on the reliability of human factors in coal mine production. In this study, we found through BN reasoning analysis that the complexity of tasks and the design of human–machine interface in smart coal mines more affect the human factor reliability of human in human–computer interaction system. This may be related to the change in the external environment of the smart coal mine and the gradual complexity of the operator's tasks.

BNs, as a directed acyclic probability map, can establish causal relationships and elucidate complex uncertainty relationships, which is beneficial for human reliability analysis. Precise inference requires access to a large amount of accurate data, which is often difficult in human reliability analysis. In this paper, in quantifying the stage dependence of cognitive errors, the decision tree proposed by SPAR-H was used in order to be able to accurately determine the situational similarity of each stage to help calculate the adjusted HEP of each node, and eventually the stage dependence of human cognitive errors between each stage was mapped to BN, making the human reliability assessment of this study more accurate.

Finally, in the process of human factors reliability modeling of intelligent coal mine hoist system, data were collected for experts in related fields. The use of triangular fuzzy numbers and the application of the collected data after processing makes the determination of model parameters more reliable and accurate. However, some uncertainties with probability distribution are difficult to avoid. Future research will explore methods to further minimize the impact of uncertainty on the model results. In addition, since the cognitive processes of operators in smart coal mine hoist systems are more similar to most of the smart coal mine HMI operational tasks, the proposed human factors reliability assessment method can be adapted to other operators in smart coal mines.

## Conclusion

Human reliability assessment of coal mine systems has been a hot topic of research. However, few studies have focused on human reliability assessment in intelligent coal mine systems, especially considering the stage dependence of human cognitive errors in phased-mission system. To address this challenge, this paper first analyzes the stage dependence of cognitive errors of hoist operators and classifies them into four types based on a cognitive-behavioral model, and constructs decision trees to quantify the degree of human cognitive dependence at each stage. Then, a new reliability analysis method for hoist operators in PMS considering the stage dependence of human cognitive errors is proposed. The reliability assessment model of hoist system human is constructed with the help of BN, which is developed to represent the causal relationship between the reliability of hoist system human and each node in each stage as well as the dependence between different stages, in conjunction with the characteristics of intelligent coal mine operations. The stage dependencies of human cognitive errors are also quantified separately in the developed BN. The hoist system human-causal reliability can be evaluated based on the CPT of all identified nodes. Finally, the effectiveness of the proposed modeling approach is illustrated with a three-stage task of a typical intelligent coal mine hoist.

Based on the forward and reverse directions of the BN theory, the probabilities of various influencing factors of human reliability in intelligent coal mine hoist systems can be accurately obtained. The results show that the interdependence of cognitive errors negatively affects the human factor reliability of the hoist system, and the complexity of the task of the operation, human–machine interface, education and training, and work load have a great impact on the human reliability in the smart coal mine hoist system. Therefore, certain targeted measures should be taken in advance to improve the human factor reliability of coal mine hoist systems. Although this paper studies a specific coal mine hoist operation example, the proposed method is considered applicable to other human-centered intelligent coal mine systems for human reliability assessment.

## Data Availability

Data can be obtained from the corresponding author upon reasonable request.
